# An In Vitro Model for Acute Myeloid Leukemia Relapse Using the SORE6 Reporter

**DOI:** 10.3390/ijms25010496

**Published:** 2023-12-29

**Authors:** Justine Lai, Chuquan Shang, Will Chen, Iyare Izevbaye, Michael P. Chu, Irwindeep Sandhu, Joseph Brandwein, Raymond Lai, Peng Wang

**Affiliations:** 1 Department of Medicine, Division of Hematology, University of Alberta, Edmonton, AB T6G 2R3, Canada; mpchu@ualberta.ca (M.P.C.); irwindee@ualberta.ca (I.S.); jbrandwe@ualberta.ca (J.B.); 2 Department of Laboratory Medicine and Pathology, University of Alberta, Edmonton, AB T6G 2R3, Canada; chuquan@ualberta.ca (C.S.); will.chen@ualberta.ca (W.C.); izevbaye@ualberta.ca (I.I.); rlai@ualberta.ca (R.L.); 3 Department of Medical Oncology, Cross Cancer Institute, Edmonton, AB T6G 2R3, Canada

**Keywords:** acute myeloid leukemia, relapse, cancer stem-like cell, SORE6 reporter

## Abstract

Many patients diagnosed with acute myeloid leukemia (AML) relapse within two years of the initial remission. The biology of AML relapse is incompletely understood, although cancer stem-like (CSL) cells have been hypothesized to be important. To test this hypothesis, we employed SORE6, a reporter designed to detect the transcriptional activity of the embryonic stem cell proteins Oct4 and Sox2, to identify/purify CSL cells in two FLT3-mutated AML cell lines. Both cell lines contained ~10% of SORE6^+^ cells in the steady state. Compared to SORE6^−^ cells, SORE6^+^ cells exhibited more characteristics of CSL cells, with significantly higher chemoresistance and rates of spheroid formation. SORE6^+^ cells had substantially higher expression of Myc and FLT3 proteins, which are drivers of SORE6 activity. Using a mixture of SORE6^−^/SORE6^+^ cells that were molecularly barcoded, we generated an in vitro study model for AML relapse. Specifically, after ‘in vitro remission’ induced by Ara-C, both cell lines regenerated after 13 ± 3 days. Barcode analysis revealed that most of the regenerated cells were derived from the original SORE6^+^ cells. Regenerated cells exhibited more CSL features than did the original SORE6^+^ cells, even though a proportion of them lost SORE6 activity. In bone marrow samples from a patient cohort, we found that relapsed blasts expressed significantly higher levels of Myc, a surrogate marker of SORE6 activity, compared to pre-treatment blasts. To conclude, using our in vitro model, we have provided evidence that CSL cells contribute to AML relapse.

## 1. Introduction

Acute myeloid leukemia (AML), defined by an accumulation of myeloblasts in the bone marrow and/or peripheral blood, is a type of highly aggressive hematologic cancer associated with a poor clinical outcome. Despite recent therapeutic advances, the five-year survival rate for AML patients receiving treatment with a curative intent is approximately 30% [1]. Refractory disease, defined as a failure to achieve complete remission, occurs in approximately 30% of all treated AML patients [2]. In patients who achieve complete remission after the initial treatment, disease relapses develop in 40–50% of those who are aged <65 years and in the vast majority of patients who are aged ≥65 years [3,4]. Treatment options for patients who develop relapses are limited; only ~10% of these patients survive >3 years after the diagnosis of relapse is made [5]. To improve the overall outlook for AML patients, new treatments for AML relapse are needed.

The molecular basis of AML relapse has not been extensively studied, partly due to the relative paucity of in vitro study models that can be readily established in conventional research laboratories. Several hypothetical models have been postulated. According to the clonal evolution model, small subclones of AML cells present at diagnosis acquire gene mutations that allow them to survive the initial chemotherapy [6]. In another model, the initial chemotherapy sends a subset of AML cells into a senescence-like state, which protects these cells during a period of dormancy that precedes relapse [7]. The cancer stem-cell model postulates the existence of a very small subset of cancer cells carrying stem-like features, such as a high level of chemoresistance, tumorigenicity and self-renewal capacity [8]. These few cells survive the initial treatment and eventually contribute to disease relapse after a period of dormancy. A modified version of the cancer stem-cell model incorporates the concept of cancer-cell plasticity, in which a subset of bulk cancer cells acquires cancer stemness [9]. A theme common to all models is that relapse AML cells exhibit a higher level of chemoresistance to the initial treatments compared to the original cell population, thereby explaining why the initial treatments are typically ineffective for treating relapse diseases. Overall, direct evidence supporting any of these models is relatively scarce. In support of the cancer stem-cell model, it was found that relapse AML cells from patient samples contained higher proportions of cancer stem cells, as quantified by using an in vivo limiting dilution assay [10]. In another study, the expression of the cancer stem-cell gene signature in AML blasts was found to correlate with a relatively high risk of disease relapse [11]. Nonetheless, this evidence is relatively indirect.

Aberrant expression of embryonic stem cell proteins such as Sox2, Oct4 and Myc has been shown in many cancer types, and the expression of these proteins in various cancer models has been found to significantly correlate with cancer stem-like (CSL) features and poor clinical outcomes [12,13,14,15,16]. The Sox/Oct4 response element (SORE6) reporter system, designed to detect the transcriptional activity of Sox2 and Oct4, has been successfully employed to identify/purify CSL cells in a few solid and hematologic cancer models [13,17,18]. Our group has recently reported that a small subset of cells in ALK-positive anaplastic large-cell lymphoma express SORE6 reporter activity [13]. This activity can be readily quantified based on the expression of green fluorescent protein (GFP), which is detectable by flow cytometry. Importantly, purified SORE6^+^ cells showed significantly more CSL features, including spheroid forming ability and chemoresistance, compared to their SORE6^−^ counterparts. The SORE6 reporter has never been used in AML studies. 

In this study, we aimed to test the importance of CSL cells in the context of AML relapse. We first asked whether the SORE6 reporter can be used to identify/purify CSL cells in AML cell lines. After the SORE6 reporter was confirmed to be useful in identifying/purifying CSL cells in AML cell lines, we leveraged the SORE6^−^/SORE6^+^ dichotomy to generate an in vitro study model that mimics key features of AML relapse. In view of the molecular diversity of AML, we focused our studies on AML cells with a specific molecular aberrancy, namely FLT3 mutations.

## 2. Results

### 2.1. SORE6 Activity Is Expressed in a Small Subset of Cells in Two FLT3-Mutated AML Cell Lines 

**Figure 1:**
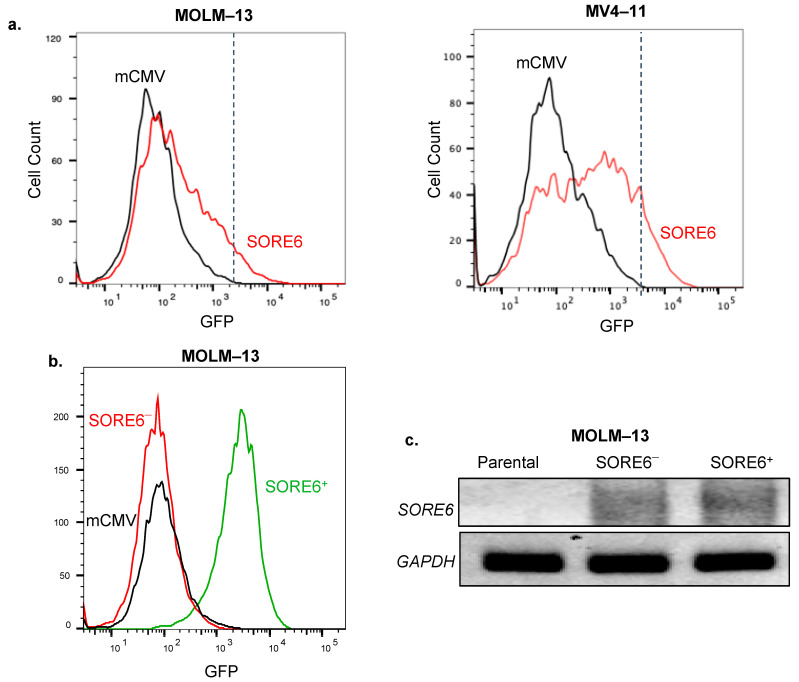
SORE6 activity is expressed in a small subset of FLT3-mutated acute myeloid leukemia (AML) cell lines. (**a**) The SORE6 reporter was transduced into two FLT3-mutated AML cell lines (MOLM–13 and MV4–11). The percentage of cells positive for green fluorescent protein (GFP) was assessed by flow cytometry seven days after the transduction. Cells transduced with mCMV were used to establish the cutoff. (**b**) MOLM–13 cells transduced with SORE6 were sorted using flow cytometry to generate a SORE6^–^ cell clone with 0.2% GFP and a SORE6^+^ cell clone with 99.0% GFP. (**c**) PCR was performed to detect the level of SORE6 integration in untransduced MOLM–13 cells, MOLM–13 SORE6^–^ cell subets, and MOLM–13 SORE6^+^ cell subsets. GAPDH was used as a loading control. SORE6 amplicons were detectable in SORE6^–^ and SORE6^+^ cell subsets, but not in untransduced cells. Original gels are shown in Figure S7a.

We first examined whether MOLM–13 and MV4–11 carry any detectable SORE6 reporter activity. Following lentiviral transduction of the reporter into these two cell lines, we found that they both included a small proportion of SORE6^+^ cells, with the expression of GFP being detectable by flow cytometry in 7% and 11% of cells, respectively (Figure 1a). Using a lentiviral vector carrying red fluorescence protein we found that the efficiency of lentiviral gene transduction for both cell lines was approximately 80% (results not shown).

We then purified SORE6^−^ and SORE6^+^ from both cell lines after they were stably transduced with the SORE6 reporter, as illustrated in Figure 1b. To confirm that the GFP-negativity in SORE6^−^ cells was not due to insufficient SORE6 integration into the genome, we performed PCR using a SORE6 primer set. As shown in Figure 1c, the SORE6 amplicons were readily detectable in both cell subsets. By performing flow cytometry every 2 weeks for a total of 8 weeks, we assessed the stability of SORE6 activity in both SORE6^−^ and SORE6^+^ cells. We found that GFP expression was consistently negative in SORE6^−^ cells from both cell lines, whereas SORE6^+^ cells derived from MOLM–13 and MV4–11 showed GFP expression in 80–95% and 75–85% of the cell population, respectively (Figure S1). 

### 2.2. SORE6^+^ Cells Have More CSL Characteristics Than SORE6^−^ Cells

**Figure 2:**
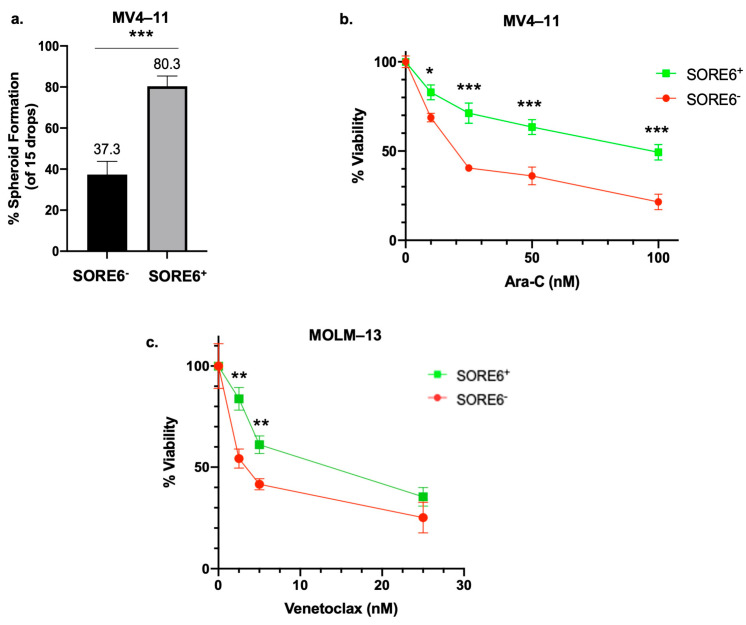
SORE6^+^ cells have more cancer stem-like features. (**a**) Percentage of spheroids formed in the hanging drop assay in MV4–11 SORE6^–^ and SORE6^+^ cell subsets. Drops were counted as a spheroid (well-defined, tightly packed, spherical cell mass) or a non-spheroid (irregularly shaped, loosely bound cell clumps). Results shown are based on three independent experiments; 15 drops were imaged in each experiment. (**b**) Cell viabilities of SORE6^–^ and SORE6^+^ cell subsets derived from a MV4–11 cell line treated with increasing doses of Ara-C for 24 h. (**c**) Cell viabilities of MOLM–13 SORE6^+^ and SORE6^–^ cells after treatment with different doses of Venetoclax (0, 5, 10 and 25 nM) in combination with low doses (10 nM) of Ara-C for 24 h. Cell viability assays (trypan blue) were performed in triplicates. Results shown as mean ± standard deviation. * *p* < 0.05, ** *p* < 0.01, *** *p* < 0.001, Student’s *t* test.

To compare SORE6^−^ and SORE6^+^ cells phenotypically, these cell subsets were subjected to the hanging drop assay [19]. As shown in Figure 2a, SORE6^+^ cells derived from MV4–11 formed a significantly greater number of spheroids compared to their SORE6^−^ counterparts (80.3% versus 37.3%, *p* < 0.001). Similar findings were observed for these two cell subsets derived from MOLM–13 (71.7% versus 31.7%, *p* = 0.006). 

We next examined whether SORE6-positivity in these two AML cell lines correlates with other CSL features such as chemoresistance. As illustrated in Figure 2b, when cells were exposed to increasing dosages of Ara-C in the presence of only 5% growth-supporting serum, SORE6^+^ cells showed significantly higher IC50 (i.e., inhibitory concentration at 50%) than SORE6^−^ cells. Specifically, the IC50 of SORE6^−^ cells derived from MV4–11 cells was 27.9 nM, as compared to 119.7 nM for SORE6^+^ cells (*p* < 0.001). The IC50 of SORE6^−^ cells derived from MOLM–13 was 32.0 nM, as compared to 87.3 nM for SORE6^+^ cells (*p* < 0.0001). 

We examined the difference in chemoresistance between the two subsets using another treatment regimen. Lose-dose Ara-C+Venotoclax (LDAC+Ven) has recently been adopted as a front-line therapy for elderly patients diagnosed with AML, including those with FLT3-mutated AML [20]. As shown in Figure 2c, in the presence of a relatively low dose of Ara-C (i.e., 10 nM), which was arbitrarily set to be 50% below the IC50 dose level of the parental cell line stably transduced with SORE6, SORE6^+^ cells were found to be significantly more resistant to Venetoclax than SORE6^−^ cells (10.9 nM versus 3.7 nM, *p* = 0.006).

### 2.3. SORE6 Activity Is Myc-Dependent 

**Figure 3:**
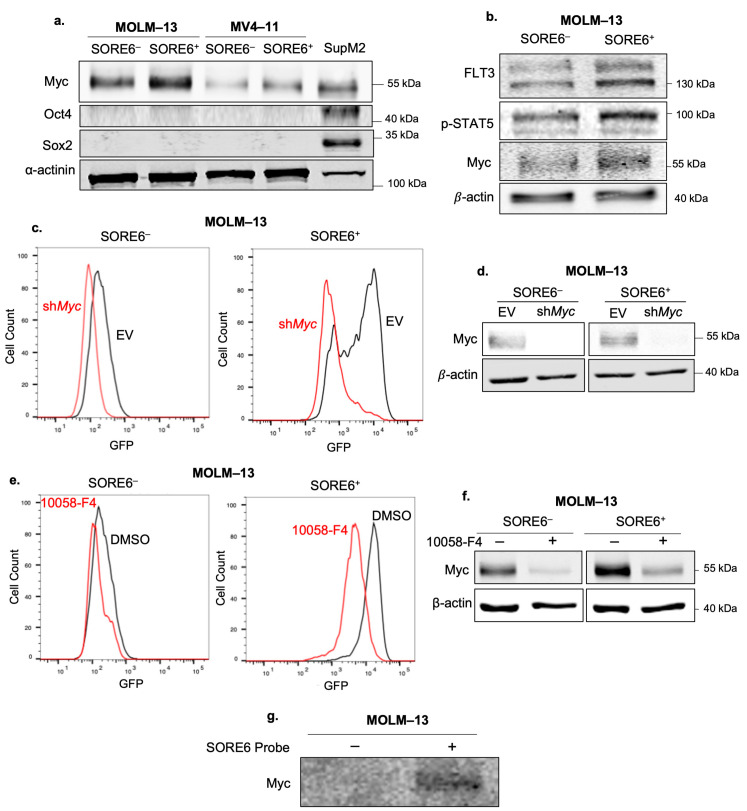
Myc is a regulator of SORE6 activity. (**a**) Expression levels of Sox2, Oct4 and Myc in SORE6^–^ and SORE6^+^ subsets derived from MOLM–13 and MV4–11 at steady state, assessed by western blots. SupM2 cells were used as a positive control for Sox2 and Oct4. Original gels are shown in Figure S7b. (**b**) Western blot analysis of *p*-STAT5, FLT3 and Myc in MOLM–13 SORE6^–^ and SORE6^+^ subsets. Original gels are shown in Figure S7c. (**c**) Flow cytometry analysis of GFP after sh*Myc* (red) in MOLM–13 SORE6^–^ and SORE6^+^ cells, with empty vector (EV) (black) used as a negative control. (**d**) Western blot to confirm the efficacy of the inhibition of Myc by shRNA. Original gels are shown in Figure S7d. (**e**) Flow cytometry analysis of GFP in SORE6^–^ and SORE6^+^ cells after treatment with a pharmacological inhibitor of Myc (10058-F4) for 24 h. Treatment with 10058-F4 (red) was compared to treatment with DMSO (black). (**f**) Western blot to confirm the efficacy of the inhibition of Myc by pharmacological inhibition with 10058-F4. Original gels are shown in Figure S7e. (**g**) DNA pull-down of Myc using a biotin-labeled SORE6 probe. A mutant DNA probe was used as the negative control. Original gels are shown in Figure S7f.

Because SORE6 was designed to detect the transcriptional activity of Sox2 and Oct4, we assessed the expression of these proteins using Western blots. In both AML cell lines, there was no/minimal Sox2 and Oct4 detected compared to SupM2, a lymphoma cell line (Figure 3a). To determine whether this low Sox2/Oct4 expression is a common phenomenon in AML, we examined the expression of Sox2/Oct4 in a panel of nine bone marrow aspirates from patients with diagnosed AML. As shown in Figure S2, all nine patient samples had minimal/no expression of Sox2/Oct4 compared to SupM2 or Jeko-1 (a mantle cell lymphoma cell line). This low Sox2/Oct4 expression in AML is consistent with the findings of previous publications [21,22]. 

Myc, another embryonic stem cell protein, was readily detectable in both SORE6^−^ as well as and SORE6^+^ cells (Figure 3a). However, the Myc level was substantially higher in SORE6^+^ cells (Figure 3b). To test whether Myc contributes directly to the relatively high SORE6 activity in SORE6^+^ cells, we inhibited Myc using shRNA. As shown in Figure 3c,d, shRNA inhibition of Myc in SORE6^+^ cells derived from MOLM–13 resulted in a dramatic reduction in the mean % of GFP^+^ cells from 72.1 to 18.7% (triplicate experiments, *p* < 0.0001). Likewise, pharmacological inhibition of Myc using 50 µM of 10058-F4 reduced the mean % of GFP^+^ cells after 24 h from 95.4% to 58.6% (*p* = 0.002) (Figure 3e). Inhibition of Myc significantly decreased the viability of both the SORE6^−^ and SORE6^+^ subsets, as shown in Figure S3. Despite the fact that 10058-F4 is known to inhibit Myc by blocking its binding to Max, we observed that the Myc protein expression decreased when cells were treated with this drug (Figure 3f). This finding is consistent with the results from other studies, which shows that Myc can upregulate its own gene expression [23,24].

Next, we asked whether overexpression of Myc in SORE6^−^ cells can increase SORE6 activity. As shown in Figure S4, no change in SORE6 activity was observed after the overexpression of Myc, suggesting that co-factors may be required for the Myc-mediated activation of SORE6 and that these co-factors are absent in SORE6^−^ cells.

To directly show that Myc is capable of binding to the Sox2/Oct4 promoter region of the SORE6 reporter, we performed a pull-down assay, using the DNA-binding consensus sequence of SORE6 as the probe. As shown in Figure 3g, SORE6 binding by Myc was observed. 

**Figure 4:**
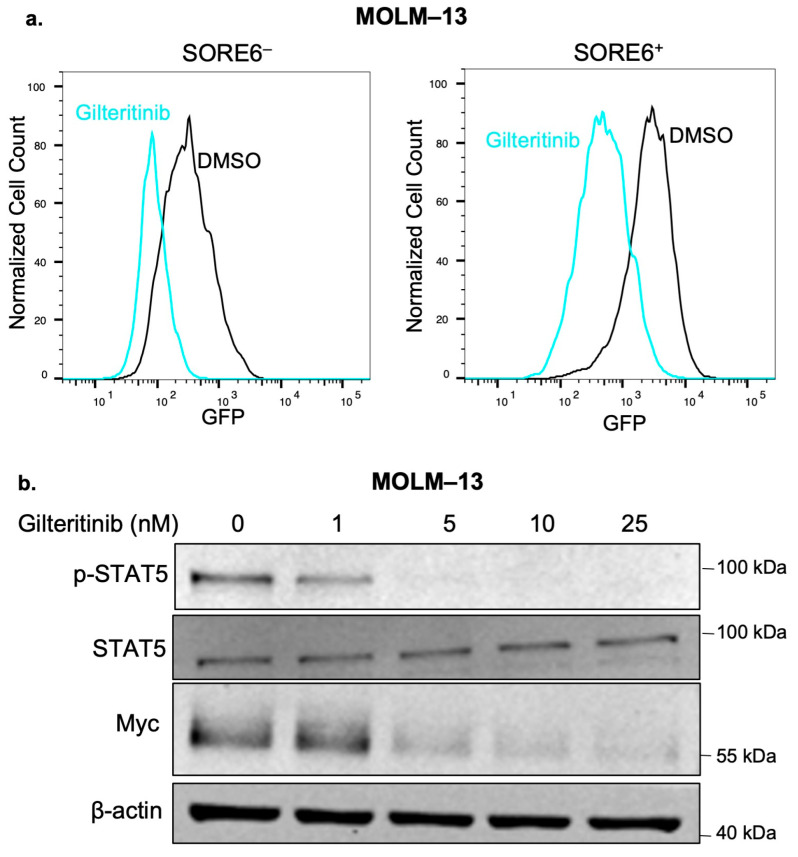
FLT3 may regulate SORE6 activity via Myc. (**a**) Flow cytometry analysis of GFP in MOLM–13 SORE6^–^ and SORE6^+^ cells after treatment with 50 nM of Gilteritinib (in blue) for 24 h compared to a DMSO control (in black). (**b**) Western blot analysis after increasing doses of Gilteritinib in MOLM–13 cells for 24 h. Downregulation of *p*-STAT5 confirmed the efficacy of inhibition of FLT3. Original gels are shown in Figure S7g.

We then asked whether the phenotypic differences between the SORE6^−^ and SORE6^+^ cells can also be attributed to differences in key cellular signaling pathways. Using Western blots, we assessed the expression of phospho-ERK, phospho-Akt, and phospho-STAT5 (p-STAT5). Except in p-STAT5, we found no appreciable differences. The level of p-STAT5 was substantially higher in SORE6^+^ cells (Figure 3b). Since previous publications have shown that FLT3 can upregulate p-STAT5 when FLT3 is mutated [25], we compared the FLT3 protein expression between the cell subsets, and we found that the FLT3 level was appreciably higher in SORE6^+^ cells. We then asked whether FLT3 contributes to SORE6 activity in SORE6^+^ cells. As shown in Figure 4a, pharmacological inhibition of FLT3 using 50 nM of Gilteritinib for 24 h significantly decreased the % of SORE6^+^ cells (80.6% versus 17.9%, *p* < 0.0001). Interestingly, Gilteritinib treatment also substantially decreased the Myc protein level (Figure 4b). The finding that Gilteritinib decreased Myc protein expression suggests that FLT3 may contribute to SORE6 activity and cancer stemness by upregulating Myc.

### 2.4. Generation of an In Vitro Model to Study AML Relapse Using SORE6^−^/SORE6^+^ Cells

**Figure 5:**
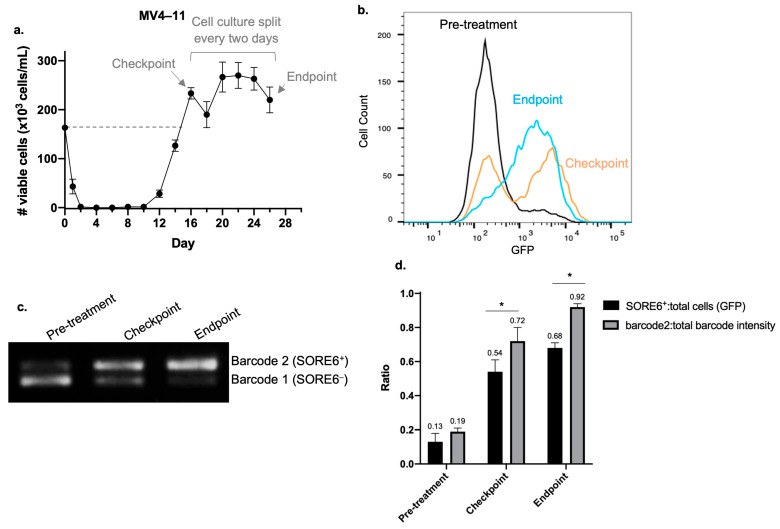
Cells that regenerated after Ara-C treatment are enriched with SORE6^+^ cells. (**a**) MV4–11 SORE6^–^ and SORE6^+^ cells pooled to a ratio of 9:1 were treated with 250 nM of Ara-C for two days. Viable cells were detected 10 days after ‘in vitro remission’ was achieved. After an additional four days, the number of viable cells returned to the number originally present at the initiation of the experiments (i.e., the checkpoint). Cells were then split every 2 days until they were harvested 10 days after the checkpoint (i.e., the endpoint). Cell-viability assays were performed in triplicate with trypan blue. (**b**) Flow cytometry analysis of GFP assessing SORE6 activity in MV4–11 cells at the pre-treatment point, the checkpoint, and the endpoint. (**c**) Relative proportion of PCR amplicons of barcode 1 and barcode 2 in MV4–11 cells at the point of pre-treatment, at the checkpoint, and at the endpoint. Original gels are shown in Figure S7h. (**d**) Comparison of the ratio of SORE6^+^:total cells assessed by flow cytometry analysis and the ratio of barcode2:total barcode intensity assessed by densitometry of PCR amplicons in cells at the point of pre-treatment, at the checkpoint, and at the endpoint. Triplicate experiments were performed. Results shown as mean±standard deviation. * *p* < 0.05, Student’s *t* test.

In view of the hypothesis that CSL cells contribute to AML relapse and our finding that SORE6^+^ cells have CSL properties, we attempted to establish an in vitro model to study AML relapse by using a mixture of SORE6^−^/SORE6^+^ cells derived from the two FLT3-mutated AML cell lines. Purified SORE6^−^ and SORE6^+^ cells derived from MOLM–13 or MV4–11 were pooled in a 9:1 ratio to approximate the ratio of these two cell subsets at the steady state. The SORE6^−^/SORE6^+^ cell mixture was treated with Ara-C to achieve the ‘in vitro remission’ state, which was defined by the absence of trypan blue-negative cells after three observations. The minimal doses of Ara-C required to induce ‘in vitro remission’ in MOLM–13 and MV4–11 were 100 nM and 250 nM, respectively. Shortly after induction of ‘in vitro remission’, we performed flow cytometry and found a mean of 300 viable cells/mL, as compared to the 150,000 cells/mL at the beginning of the experiments (i.e., 0.2% viability). A mean of 13 days after the ‘in vitro remission’ state was achieved, viable cells (i.e., trypan blue-negative) were detectable (Figure 5a). Between 3–5 days after viable cells were detectable, the number of cells reached the original number counted at the initiation of the experiments (labeled as the checkpoint). The endpoint of this experiment was arbitrarily set as 10 days after the checkpoint. 

If the hypothesis that CSL cells contribute to AML relapse is correct, we expect that SORE6^+^ cells will be the predominant cell type in the regenerated cell population. Because there is a possibility that SORE6^+^ cells in the regenerated population may originate from SORE6^−^ cells (i.e., conversion due to cancer plasticity), we repeated the experiments using SORE6^−^ and SORE6^+^ cells that had been separately molecularly barcoded. Triplicate experiments were performed using both AML cell lines, and representative results from MV4–11 are shown. As shown in Figure 5b, at the checkpoint, the mean %SORE6^+^ cells measured by flow cytometry was 54.1 ± 6.9%, compared to 13.0 ± 4.7% before Ara-C treatment. At the endpoint, the mean %SORE6^+^ was 68.0 ± 3.0%. Then, using PCR to detect the molecular barcodes, we assessed the ratio of the original SORE6^−^ cells to SORE6^+^ cells present in the regenerated cell population. As shown in Figure 5c, barcode 2 (top band), which was used to label the original SORE6^+^ cells, was a relatively weak band compared to the band associated with barcode 1 (lower band, original SORE6^−^ cells) at the beginning of the experiment. In contrast, barcode 2 was slightly stronger than barcode 1 at the checkpoint. Using densitometry, the mean barcode2:total barcode intensity (barcode1 + barcode2) ratio at the beginning of the experiment was 0.19 ± 0.02, compared to 0.72 ± 0.08 at the checkpoint and 0.92 ± 0.02 at the endpoint. As shown in Figure 5d, flow cytometry revealed that the ratios of SORE6^+^:total cells at the checkpoint and endpoint were significantly lower than the barcode2:total band intensity ratios at these two time points, suggesting that a small subset of SORE6^+^ cells had converted/differentiated during the course of these experiments. Similar experiments were repeated using MOLM–13 cells, and similar results were obtained (Figure S5).

We next asked whether the regenerated cells at the endpoint possessed more CSL features. Specifically, they are significantly more resistant to Ara-C compared to untreated SORE6^+^ cells (IC50 140.2 versus 98.9 nM, *p* = 0.007). Regenerated cells at the endpoint also formed a significantly greater number of spheroids in the hanging drop assays compared to SORE6^+^ cells (88.3 versus 68.0%, *p* < 0.001). These results suggest that the regenerated cells had acquired a significantly stronger CSL phenotype despite the occurrence of conversion into SORE6^−^ cells in a small proportion of cells. 

### 2.5. Samples from Relapsed Patient Have More Stem-like Cells Than Samples from Initial-Diagnosis Patients 

**Figure 6:**
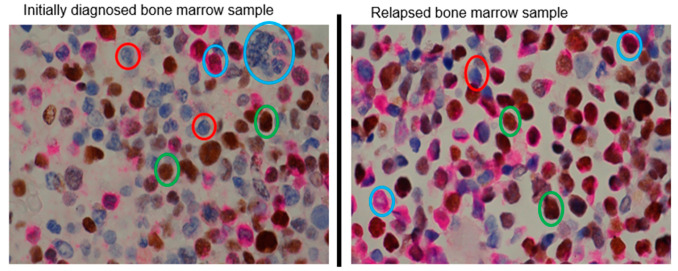
Bone marrow samples from relapsed patients have a higher percentage of Myc-positive cells compared to bone marrow samples from patients at the point of initial diagnosis. Double immunostaining of Myc (brown) and myeloperoxidase (pink) was performed in paired AML bone marrow clot sections from the time of initial diagnosis and after relapse from the same patient (400× magnification). Blasts were identified by their morphology and by weak/negative myeloperoxidase expression. Blasts were counted as either being Myc-positive (example circled in green) or Myc-negative (example circled in red). Examples of non-blast cells are circled in blue. For this patient, the mean % of Myc-positive blasts was 87.7% in the samples from after relapse, compared to 49.2% Myc-positive blasts in the sample taken at the time of initial diagnosis (*p* < 0.0001).

Lastly, we aimed to collect evidence to support the importance of SORE6^+^ cells in clinical samples. As Myc is a key driver of SORE6 activity and has previously been shown to regulate cancer stemness [12], we used Myc protein expression as a marker of cancer stemness. We examined the Myc protein level using western blots and AML bone marrow samples from a patient cohort, including nine samples from patients with an initial diagnosis and nine from relapsed patients. We quantified Myc expression with densitometry analysis and normalized the values to the blast counts for each case. Overall, samples from relapsed patients had significantly higher levels of Myc expression, with a 16.4-fold upregulation in the mean Myc expression level in the relapsed group compared to the initial-diagnosis group (*p* = 0.04) (Table 1). Additionally, immunostaining of Myc was performed on bone marrow clot sections from a patient with samples taken at both initial diagnosis and relapse. In order to minimize the inclusion of early myeloid precursors in the evaluation, we performed double immunostaining using Myc and myeloperoxidase, and strongly myeloperoxidase-positive cells were excluded from being counted as blasts. As illustrated in Figure 6, we found that Myc-positive blasts were present in significantly greater numbers in the bone marrow sample taken after relapse. 

## 3. Discussion

Disease relapse poses one of the most important clinical challenges in treating AML patients. Concepts related to clonal evolution, senescence, and cancer stem cells/CSL cells have been postulated to be potentially important. Nonetheless, due to the paucity of study models, evidence supporting the relevance of these concepts is relatively scarce and circumstantial. Thus, one of our main research goals was to establish an in vitro experimental model that mimics key characteristics of AML relapse. Our in vitro model is primarily based on the use of the SORE6 reporter to identify/purify CSL cells. The substantial duration of limited cell proliferation during the Ara-C-induced ‘in vitro remission’ and the subsequent regeneration mimic disease remission and relapse of AML, respectively. The relatively high chemoresistance in the regenerated cell population also mirrors the clinical observation that relapsed diseases are highly resistant to frontline chemotherapeutic agents such as Ara-C. This study model can be readily established in a conventional research laboratory, so long as SORE6^−^/SORE6^+^ cell clones can be obtained/generated. 

The concept that the reporter activity of SORE6 correlates with cancer stemness has been demonstrated in a number of solid tumors and hematologic cancers [13,17,18,26]. Common themes of all published SORE6 studies are as follows: (1) only a minority of cells (~2–20%) in various cancer cell lines were identified as SORE6^+^ at the steady state and (2) SORE6^+^ cells were characterized by more CSL features, including tumorigenicity and chemoresistance, compared to SORE6^−^ cells. The design of SORE6 was built on the assumption that cancer stemness is associated with the transcriptional activity of two embryonic stem cell proteins, Sox2 and Oct4. This approach is in contrast with that used in most of the previously published studies on cancer stemness, which are based on the expression of specific cell-surface proteins with no known functional link to cancer stemness. Experimental inhibition of Sox2, Oct4 or Myc can lead to significant decreases in the CSL phenotypes in cancer cells [12,13,27,28]. To our knowledge, this study is the first to use the SORE6 reporter to study AML. Our finding that SORE6 activity significantly correlates with cancer stemness is in line with the findings of other SORE6 studies. In contrast with a small number of other SORE6 studies, we found that Myc, rather than Sox2 or Oct4, is the key regulator of SORE6 reporter activity in FLT3-mutated AML cells. Similar findings were observed in a study examining triple-negative breast cancer using a molecular reporter similar to SORE6, the SRR2 reporter, which detects the transcriptional activity of Sox2. In this study, 16 different transcription factors, including Myc, were found to bind to the SRR2 consensus sequence [12]. Thus, the redundancy of these consensus sequences may explain why SORE6 activity is observed despite the lack of detectable Sox2/Oct4 expression in AML cells. 

In our study, we found that Myc is capable of binding to the DNA-binding consensus sequence of SORE6, supporting the role of Myc as an activator of the SORE6 reporter. Even though SORE6^−^ cells had lower levels of Myc expression compared to SORE6^+^ cells, Myc expression was detectable in the SORE6^−^ subset. The fact that no appreciable SORE6 activity was detected in this cell subset may be explained by the following possibilities: (1) SORE6 activity may require a threshold of Myc protein expression, and the Myc expression in SORE6^−^ cells is below the threshold, and/or (2) SORE6 reporter activity requires co-activators of Myc, and these coactivators are absent in SORE6^−^ cells. In support of the latter theory, we have found that Myc overexpression in SORE6^−^ cells did not induce an increase in SORE6 activity. This observation is in parallel with the results of our prior studies using a Sox2 reporter (SRR2) in breast cancer cells, wherein no reporter activity was observed in the SRR2-negative subset after Sox2 overexpression [29].

Leveraging molecular barcoding, we directly addressed the question of whether CSL cells are major contributors to AML relapses. Analysis of the barcoded SORE6^−^ and SORE6^+^ cells after regeneration provided evidence that CSL cells are the major contributors to AML relapses. Firstly, while SORE6^+^ cells accounted for approximately 10% of the cell population at the beginning of the experiments, the proportion of original SORE6^+^ cells increased to approximately 70% and 90% at the checkpoint and at the endpoint, respectively. The progressive increase in the proportion of SORE6^+^ cells during the in vitro relapse suggests that most of the SORE6^−^ cells that emerged after the regeneration did not survive to the endpoint. Based on this observation, it is tempting to hypothesize that AML relapse may involve two distinct phases: (1) the initial emergence from the ‘in vitro remission’ condition, and (2) a subsequent clonal expansion of cell clones that are primarily derivatives of SORE6^+^ cells.

The term ‘cancer plasticity’ has been used to describe the ability of cancer cells to acquire stemness [30]. One example is epithelial-mesenchymal transition (EMT), in which exposure of epithelial cells to various insults can change their morphology to that of mesenchymal cells, a change that is accompanied by an increase in chemoresistance and the aberrant expression of various embryonic stem cell proteins [31,32,33]. Using a Sox2 reporter (i.e., SRR2), our group has previously demonstrated the acquisition of stemness in breast cancer and lymphoma cells exposed to oxidative stress [12,34]. We asked whether cancer plasticity occurs in our AML model. If a substantial degree of cancer plasticity occurred, the %SORE6^+^ cells (by flow cytometry) in the regenerated cell population should be appreciably higher than the % of cells derived from the original SORE6^+^ cells (as determined by barcoding). Our finding that the derivatives of the original SORE6^+^ cells account for >90% of cells at the endpoint (as determined by barcoding) argues against the importance of cancer plasticity in this model. However, based on the observation that the %SORE6^+^ cells (by GFP) was consistently lower than the % of original SORE6^+^ cells (as determined by barcoding), substantial differentiation may have occurred. This phenomenon is not too surprising to us, as we believe that SORE6^+^ cells are CSL and thus should be capable of self-regeneration/differentiation. Interestingly, when tracking the stability of the SORE6^+^ subset over time (8 weeks), we observed that SORE6^+^ subsets were stable and that no differentiation occurred. We speculate that the lack of phenotypic conversion from SORE6^+^ to SORE6^−^ subsets may be due to the absence of certain factors, such as microenvironmental factors. Identifying the factors facilitating phenotypic conversion can be a topic to be investigated in future studies.

In conclusion, we described the establishment of an in vitro model for AML relapse based on the SORE6^−^/SORE6^+^ dichotomy. This model has characteristics that mimic the clinical features of AML relapse. Using this model, we have provided evidence to support the importance of CSL cells in AML relapse after Ara-C treatment. Further research using this model may facilitate the identification of cellular pathways and therapeutic targets for AML relapse.

## 4. Materials and Methods

### 4.1. Cell Culture 

Two FLT3-mutated AML cell lines, MV4–11 and MOLM–13 [35], were cultured in Roswell Park Memorial Institute (RPMI) 1640 (Invitrogen, Waltham, MA, USA) supplemented with 10% fetal bovine serum (Gibco, Waltham, MA, USA) and 1% penicillin/streptomycin (Gibco, Waltham, MA, USA). Cells transduced with the SORE6 reporter, carrying a puromycin selection marker, were cultured in the presence of 0.25 µg/mL puromycin (Gibco, Waltham, MA, USA).

### 4.2. Generation of SORE6^−^ and SORE6^+^ Subsets

The SORE6-mCMVo-dsCop-GFP-PURO (SORE6) reporter and mCMVp-dsCopGFP-PURO (mCMV) plasmids were kind gifts from Dr. Lalage Wakefield (National Cancer Institute, Bethesda, MD, USA) [17]. AML cell lines underwent lentiviral transduction with these plasmids, as described previously [36].

SORE6 activity was quantified by measuring the level of GFP expression using flow cytometry. Cells transduced with mCMV were used to establish the cutoff. SORE6^−^ and SORE6^+^ cell subsets were generated using flow cytometric cell sorting (Sony MA900, New York, NY, USA). Cells expressing relatively high levels of GFP (i.e., top ~4–8%) were purified and cultured into the SORE6^+^ clone, and cells expressing no detectable GFP (i.e., the bottom ~4–8%) were purified and cultured into the SORE6^−^ clone. All experiments performed in this study employed SORE6^+^ cells showing ≥75% GFP positivity and SORE6^−^ cells showing <5% GFP detectable by flow cytometry.

### 4.3. Antibodies, Plasmids, and Drug Treatments 

Primary antibodies used in western blot studies included anti-MYC (Y69, #ab32072), anti-Sox2 (EPR3131; #ab92494) and anti-Oct4 (#ab19857), which were purchased from Abcam (Cambridge, MA, USA), anti-β-actin (#sc-47778) from Santa Cruz Biotechnology (Santa Cruz, CA, USA), anti-FLT3 (8F2, #3462) from Cell Signaling (Danvers, MA, USA), and anti-p-STAT5 (Tyr694, #9359) from Cell Signaling. Additionally, anti-myeloperoxidase (A1F4; #MA5-42397) from Invitrogen (Waltham, MA, USA) and the anti-MYC mentioned above were used in immunostaining experiments. Gilteritinib (ASP2215, #S7754) was purchased from Selleckchem (Houston, TX, USA), and Ara-C (PHR1787) was purchased from Sigma-Aldrich (St Louis, MO, USA). Venetoclax was gifted by Abbvie. Short hairpin RNA (shRNA) plasmid for Myc was purchased from MilliporeSigma (Burlington, MA, USA). Myc overexpression vector was purchased from Addgene (#190618) (Watertown, MA, USA). 

### 4.4. Polymerase Chain Reaction

Genomic DNA extraction was performed using PureLink^®^ Genomic DNA mini kit (Invitrogen, Waltham, MA, USA), using a procedure based on the manufacturer’s protocol. PCR reactions to detect cell barcoding were performed using the CloneTracker 4-barcode-plus cell labeling kit (Cellecta, Mountain View, CA, USA), following the manufacturer’s protocol. PCR reactions to detect SORE6 were done using SYBR Green Real-Time PCR Master Mixes, as indicated by the manufacturer (ThermoFisher Scientific, Waltham, MA, USA). Bands were visualized by gel electrophoresis with a 3% agarose gel. Primer sequences were as follows:

SORE6: F-5′-ACAATGGCCTTGGTGCAG-3′ & R-5′-TGCACCAAGGCCATTGTAA-3′; GAPDH: F-5-GGTCTCCTCTGACTTCAACAGCG-3 & R-5-ACCACCCTGTTGCTGTAGCCAA-3.

### 4.5. Hanging Drop Assay

For this assay, 100,000 cells were seeded in 1 mL of culture media. Next, 10 µL drops were pipetted onto the lid of a cell culture dish. Spheroid formation was assessed after 48 h using a light microscope. The criterion for spheroid formation was viable cells were clustered together in a sphere. Every well contained one of these two types of cell clumps: (1) true spheroid, which is a well-defined, tightly packed, spherical cell mass (Figure S6a) or (2) irregularly shaped, loosely bound cell clumps (Figure S6b). On high magnification, the true spheroids consist of viable-appearing cells, as shown by their refractile appearance, as opposed to cell debris in the non-viable cell clumps. A total of 15 drops were randomly selected for analysis per plate. 

### 4.6. Cell Viability Assay

Cells were plated at a concentration of 250,000 cells/mL of media with 5% fetal bovine serum. Cell viability was assessed using trypan blue exclusion. IC50 was calculated using GraphPad Prism version 8 software (GraphPad Software, San Diego, CA, USA).

### 4.7. Western Blot

Cell pellets were lysed with RIPA buffer (MilliporeSigma, Burlington, MA, USA), with protease and phosphatase inhibitors (MilliporeSigma, Burlington, MA, USA). Proteins were separated on a 10–15% polyacrylamide SDS-PAGE gel, transferred to a nitrocellulose membrane (GE Healthcare, Velizy-Villacoublay, France), and then incubated with primary antibodies. The membrane was then incubated with horseradish peroxidase-conjugated secondary antibodies. Bands were visualized with an Odyssey^®^ Infrared Imaging System (LI-COR, Lincoln, NE, USA). Triplicate experiments were conducted for all western blots. For all blots, changes to contrast/intensity were applied to every pixel and were carried out using Microsoft^®^ PowerPoint version 16.77. The original gels are shown in Figure S7. 

### 4.8. DNA Pull-Down Assay

DNA pull-down was performed as described previously [13]. The sequence of the SORE6 probe was as follows: 

5′-BiosgCCCTTTTGCATTACAATGTCTTTTGCATTACAATGTCTTTTGCATTACAATG-3′. A mutant DNA probe was used as the negative control. 

### 4.9. Barcode Labeling of SORE6 Sorted Cells

The CloneTracker 4-barcode-plus cell labeling kit (Cellecta, Mountain View, CA, USA) was employed to track cell fates. Barcodes were transduced into purified SORE6^+^ and SORE6^−^ cells, following the manufacturer’s protocol. In this manuscript, the barcode used for SORE6^−^ cells was termed “barcode 1”, while that for SORE6^+^ cells was termed “barcode 2”. The unique sequence in each barcode was detectable by polymerase chain reaction and a set of primers supplied by Cellecta (Mountain View, CA, USA). Both barcodes also carried a red fluorescence protein to allow the identification of cells that had undergone successful transduction of the barcodes. The barcodes were stably expressed in both cell populations over the 4 weeks of our experiments, with >95% of cells expressing red fluorescence protein that was detectable by flow cytometry.

### 4.10. In Vitro AML Relapse Model

MOLM–13 and MV4–11 cells were plated to a concentration of 150,000 cells/mL. Cells were treated with Ara-C for two days, at which point medium without Ara-C was added to the culture. The lowest doses that induced ‘in vitro remission’, defined by the absence of trypan blue-negative cells after three observations, were found to be 100 nM for MOLM–13 and 250 nM for MV4–11. Immediately after the induction of ‘in vitro remission’, we performed flow cytometry and quantified the number of viable cells. Using the forward-scatter/size-scatter gating strategy, we found a median of ~300 viable cells/mL (i.e., ~0.2% viability). To detect regeneration, 200 µL of the cell culture was removed for cell counting with trypan blue every two days, and the cell culture was replenished with 200 µL of fresh culture medium. Once the cell density reached the original density (labeled as the ‘checkpoint’), the cell culture was split in half and topped up with fresh culture medium every two days.

### 4.11. Patient Samples

Nine AML bone marrow aspirates from patients at the point of initial diagnosis and nine samples from relapsed patients were retrieved retrospectively from the University of Alberta Hospital. Formalin-fixed/paraffin-embedded bone marrow clot sections representing the initial-diagnosis sample, as well as relapse samples from the same single FLT3-mutated AML patient, was also retrieved retrospectively from the University of Alberta Hospital. The use of these patient samples was approved by the Health Research Ethics Board of Alberta (HREBA.CC-21-0253).

### 4.12. Immunohistochemistry

After deparaffinization and rehydration of the tissue, antigen retrieval was performed using an EDTA buffer (Sigma-Aldrich, St Louis, MO, USA). Slides were incubated overnight at 4 °C with an anti-Myc antibody (Abcam, Cambridge, MA, USA). Sections were blocked with 3% hydrogen peroxide and then incubated with Dako EnVision+ System HRP Labelled Polymer secondary antibody (Agilent, Santa Clara, CA, USA) at room temperature for one hour. Dako DAB+ Chromagen (Agilent) was used to develop the sections. Antigen retrieval was then repeated using citrate buffer (Sigma-Aldrich, St Louis, MO, USA). Sections were incubated with anti-myeloperoxidase antibody (Invitrogen, Waltham, MA, USA) overnight at 4 °C. After secondary antibody incubation with Dako EnVision+ System HRP Labelled Polymer, slides were developed with AEC peroxidase (Enzo Life Sciences, ENZ-43825, Farmingdale, NY, USA). Slides were coverslipped with an aqueous mounting medium. To score the slides, blasts were identified as being myeloperoxidase-weak/negative and by their morphology. Areas with predominantly immature cells relatively devoid of erythroid cells were chosen for evaluation. Megakaryocytes were similarly excluded based on their morphology.

### 4.13. Statistical Analysis

Statistical analyses were performed using GraphPad Prism 8 (Graphpad Software Inc., LaJolla, Ca, USA). Densitometry analysis of western blots was performed using ImageJ version 1.53a (U.S. National Institutes of Health, Bethesda, MD, USA). Finally, *p*-values were calculated using two-tailed Student’s *t*-tests.

## Figures and Tables

**Table 1:** Bone marrow samples from relapsed patients exhibit higher Myc expression compared to bone marrow samples from patients at the point of initial diagnosis. Data from densitometry analysis for nine of each type of bone marrow aspirate.

Initially Diagnosed Sample #	Densitometry	Blast Count (%)	Densitometry Normalized to Blast Count	Relapsed Sample #	Densitometry	Blast Count (%)	Densitometry Normalized to Blast Count
1	0.0	60	0.0	1	7.6	13	58.1
2	0.0	21	0.0	2	3.6	33	10.9
3	0.0	23	0.0	3	3.4	21	16.4
4	0.0	36	0.0	4	0.8	7	11.5
5	0.0	41	0.0	5	0.0	7	0.0
6	0.7	24	2.9	6	10.0	14	71.5
7	4.7	34	13.8	7	15.6	14	111.1
8	0.0	20	0.0	8	0.0	24	0.0
9	0.0	80	0.0	9	0.0	10	0.0
		**Mean**	1.9			**Mean**	31.1
		**SD**	4.6			**SD**	39.8

Relapsed/initially diagnosed: 16.4. *p* = 0.04.

## Data Availability

Data are contained within the article and Supplementary Materials.

## Supplementary Materials

The following supporting information can be downloaded at: https://www.mdpi.com/article/10.3390/ijms25010496/s1.
