# Supplementary material for: An In Vitro Model for Acute Myeloid Leukemia Relapse Using the SORE6 Reporter
Source: Int J Mol Sci. 2023 Dec 29;25(1):496. doi: 10.3390/ijms25010496 (PMC10779023; doi:10.3390/ijms25010496)
Supplement: Supplementary file 1 [file ijms-25-00496-s001.zip › ijms-2699775-supplementary.pdf]

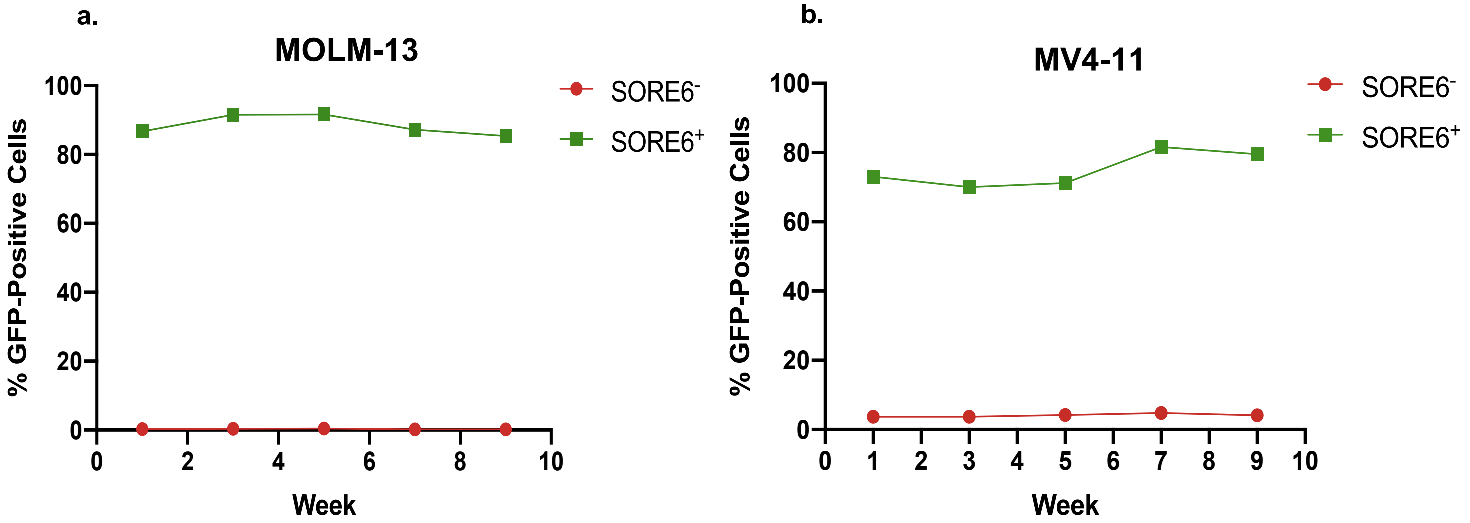

Figure S1. SORE6^{-} and SORE6^{+} subsets over time. Percentage of SORE6^{+} cells measured by FACS analysis of GFP in SORE6^{-} and SORE6^{+} clones in (a) MOLM-13 and (b) MV4-11 cells. GFP was analyzed over a period of 8 weeks, starting at one week post transfection. Cells transfected with mCMV were used as a control.

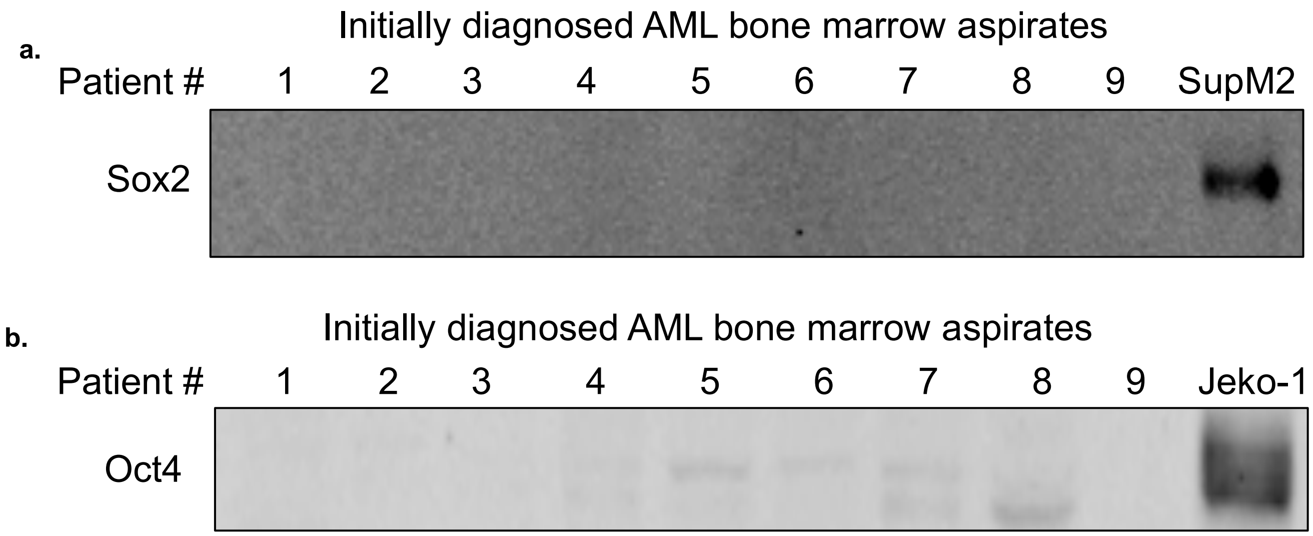

Figure S2. Sox2 and Oct4 are absent in AML patient samples. Protein expression level of (a) Sox2 and (b) Oct4 in nine initially diagnosed AML bone marrow aspirates, assessed by western blots. SupM2 cells were used as a positive control for Sox2, and Jeko-1 cells were used as a positive control for Oct4.

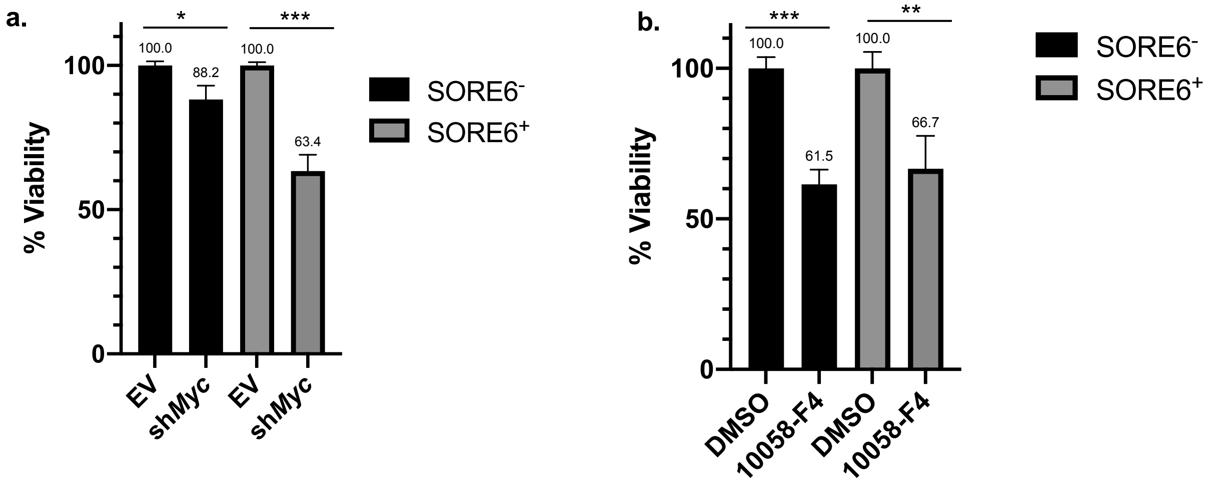

Figure S3. Cell viability after Myc inhibition. (a) Cell viability (Trypan blue) after knockdown of Myc using shRNA in MOLM-13 SORE6$^-$ and SORE6$^+$ subsets. Cells were counted 48 hours after the second transduction with sh*Myc*. Cell viability was normalized to cells transduced with an empty vector. (b) Cell viability of MOLM-13 SORE6$^-$ and SORE6$^+$ subsets after treatment with 50 μM of the Myc inhibitor 10058-F4 for 24 hours, normalized to cells treated with DMSO. All measurements based on triplicate experiments. Results shown as mean ± standard deviation. * $p < 0.05$, ** $p < 0.01$, *** $p < 0.001$, Student's t test.

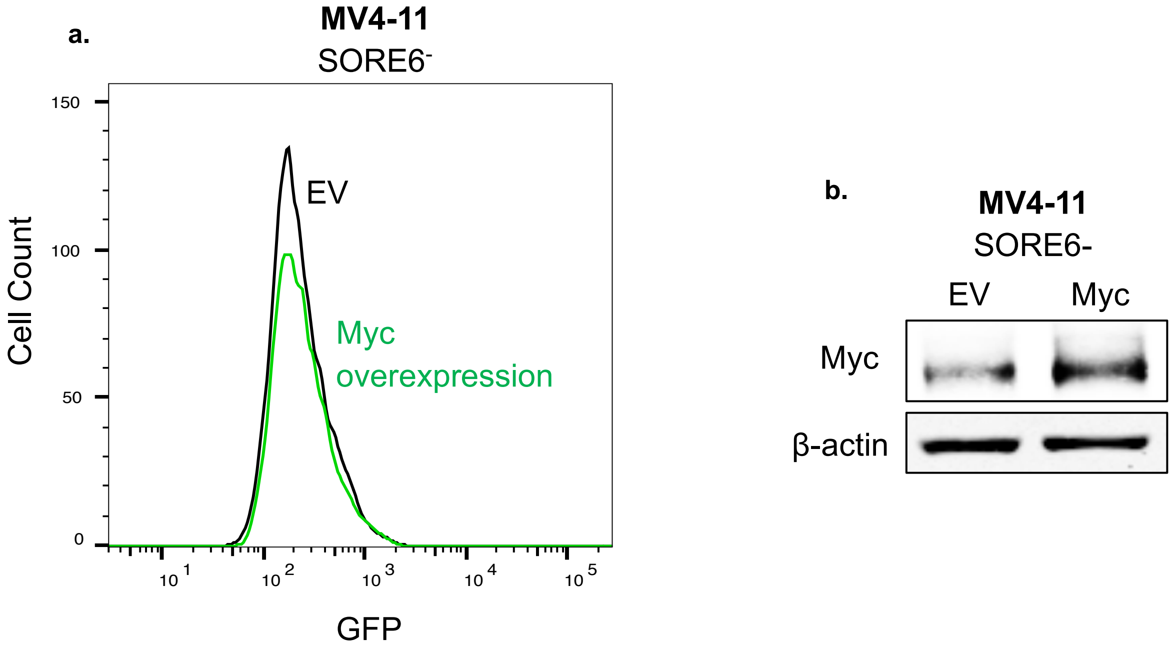

Figure S4. Myc overexpression does not change SORE6 expression in SORE6$^-$ cells. (a) FACS analysis of GFP after *Myc* overexpression (green) in MV4-11 SORE6$^-$ cells, with empty vector (EV) (black) used as a negative control. (b) Western blot to confirm the efficacy of *Myc* overexpression by lentiviral transduction.

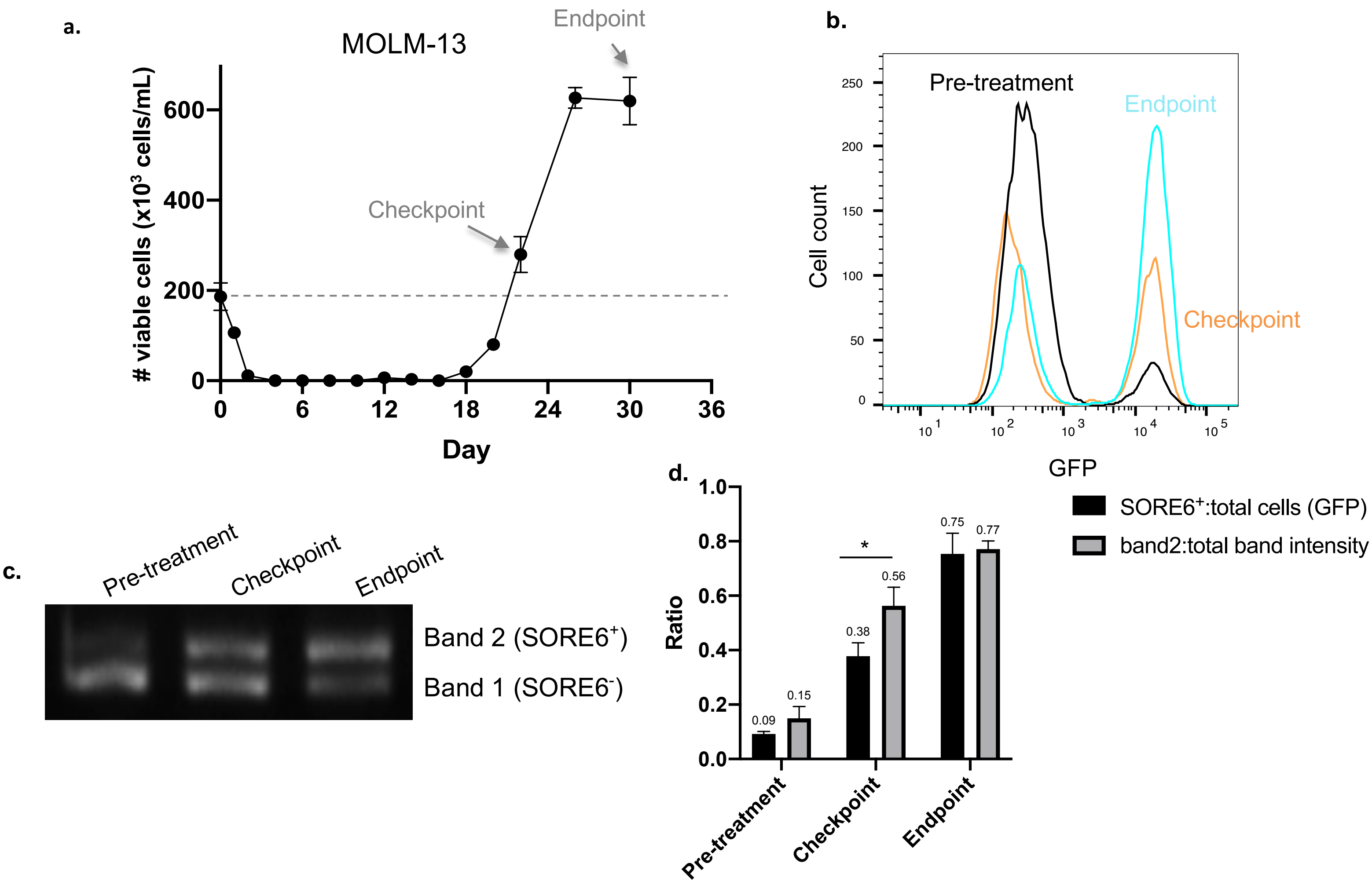

Figure S5. MOLM-13 cells regenerated after Ara-C treatment are enriched with SORE6$^+$ cells. (a) MOLM-13 SORE6$^-$ and SORE6$^+$ cells pooled to a ratio of 9:1 were treated with 100 nM of Ara-C for two days. Viable cells were detected 14 days after '*in-vitro* remission' was achieved. After an additional six days, the number of viable cells returned to the original number at the initiation of the experiments (i.e. checkpoint). Cells were also harvested 10 days after the checkpoint (i.e. endpoint). Cell viability assays were performed in triplicates and assessed by Trypan Blue. (b) FACS analysis of GFP assessing SORE6 activity in MOLM-13 cells at pre-treatment, checkpoint, and endpoint. (c) Relative proportion of PCR amplicons of barcode 1 and barcode 2 in MOLM-13 cells at pre-treatment, checkpoint, and endpoint. (d) Comparison of SORE6$^+$:total cell ratio assessed by FACS analysis and barcode2:total band intensity ratio by densitometry of PCR amplicons in cells at pre-treatment, checkpoint, and endpoint. Triplicate experiments were performed. Results shown as mean±standard deviation. * $p < 0.05$, ** $p < 0.01$, *** $p < 0.001$, Student's t test.

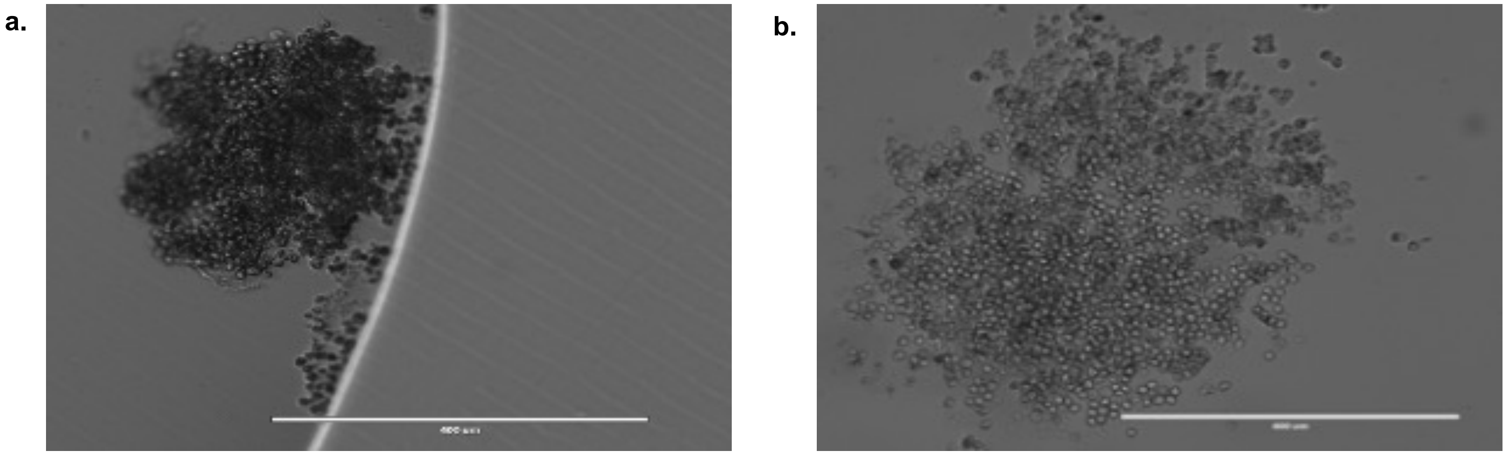

Figure S6. Image of a spheroid and non-spheroid in the hanging drop assay. (a) Example of a true spheroid with a well-defined, tightly packed spherical cell mass. (b) Example of a non-spheroid, with loosely bound cell clumps. Imaged with a bright-field microscope (40x).

a. Figure 1c.

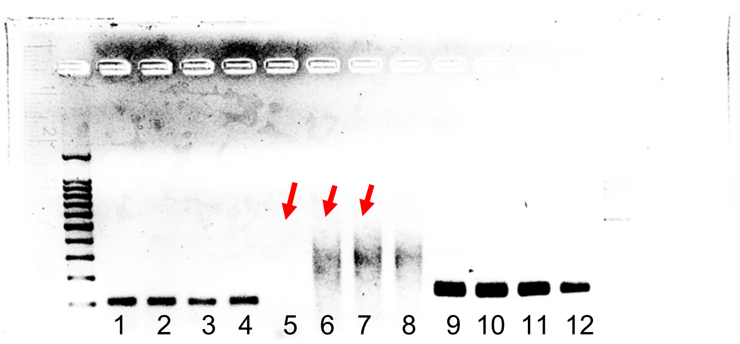

1. MOLM-13 Parental Cells, Myc primer
2. MOLM-13 SORE6^{-} Cells, Myc primer
3. MOLM-13 SORE6^{+} Cells, Myc primer
4. **MOLM-13 Cells transfected with SORE6 (not sorted), Myc primer**
5. **MOLM-13 Parental Cells, SORE6 primer**
6. **MOLM-13 SORE6^{-} Cells, SORE6 primer**
7. MOLM-13 SORE6^{+} Cells, SORE6 primer
8. MOLM-13 Cells transfected with SORE6 (not sorted), SORE6 primer
9. MOLM-13 Parental Cells, GAPDH primer
10. MOLM-13 SORE6^{-} Cells, GAPDH primer
11. MOLM-13 SORE6^{+} Cells, GAPDH primer
12. MOLM-13 Cells transfected with SORE6 (not sorted), GAPDH primer

b. Figure 3a.

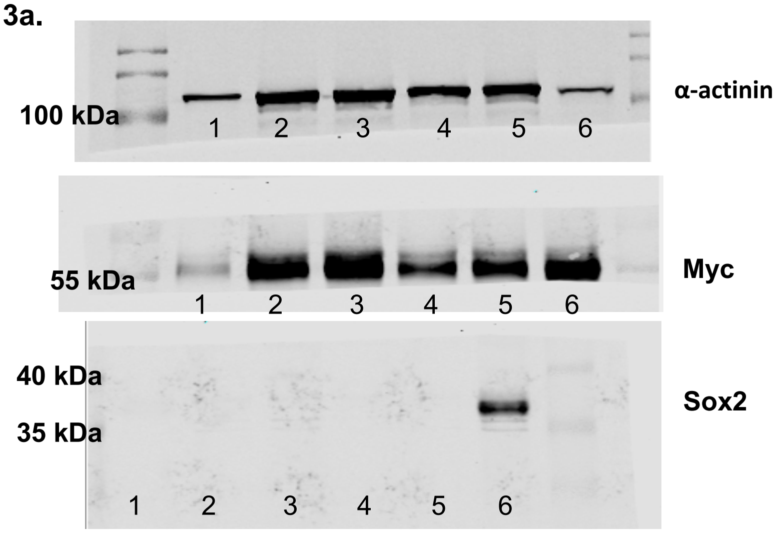

1. 293T cells
2. **MOLM-13 SORE6^{-}**
3. **MOLM-13 SORE6^{+}**
4. **MV4-11 SORE6^{-}**
5. **MV4-11 SORE6^{+}**
6. **SupM2**

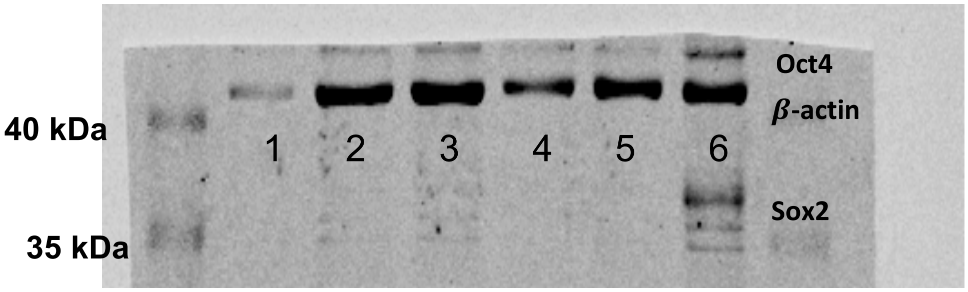

c. Figure 3b.

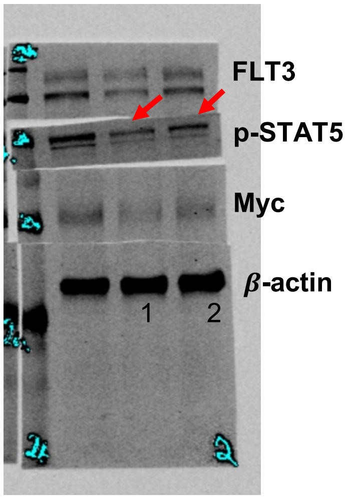

1. MOLM SORE6$^-$
2. MOLM SORE6$^+$

d. Figure 3d.

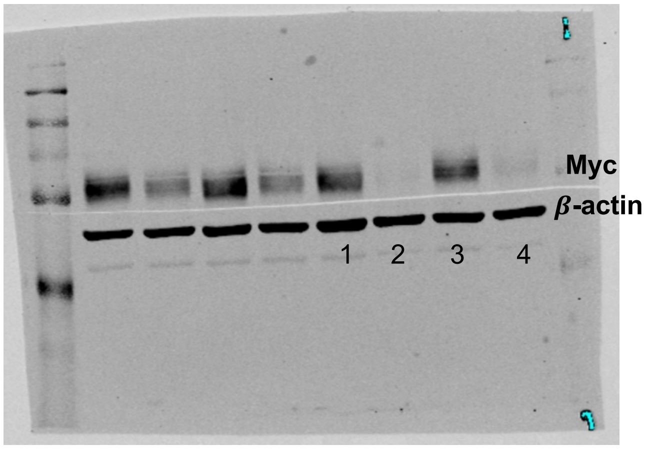

1. MOLM-13 SORE6$^-$ empty vector
2. MOLM-13 SORE6$^-$ sh*Myc*
3. MOLM-13 SORE6$^+$ empty vector
4. MOLM-13 SORE6$^+$ sh*Myc*

e. Figure 3f.

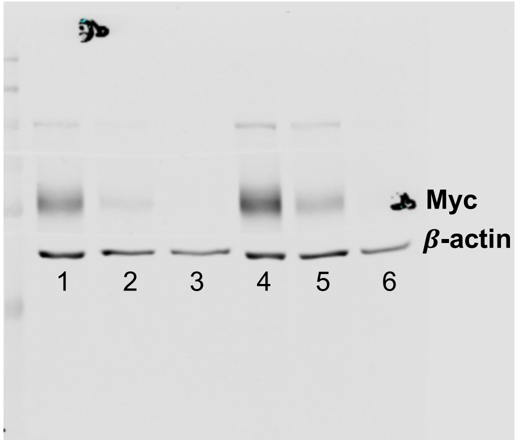

1. **MOLM-13 SORE6$^-$ DMSO**
2. **MOLM-13 SORE6$^-$ 50 μM 10058-F4**
3. MOLM-13 SORE6$^-$ 100 μM 10058-F4
4. **MOLM-13 SORE6$^+$ DMSO**
5. **MOLM-13 SORE6$^+$ 50 μM 10058-F4**
6. MOLM-13 SORE6$^+$ 100 μM 10058-F4

f. Figure 3g.

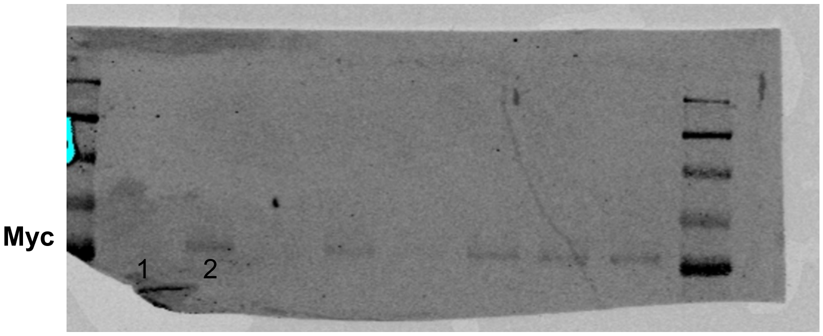

1. MOLM-13 parental – mutant pulldown
2. MOLM-13 parental – SORE6 pulldown

g. Figure 4b.

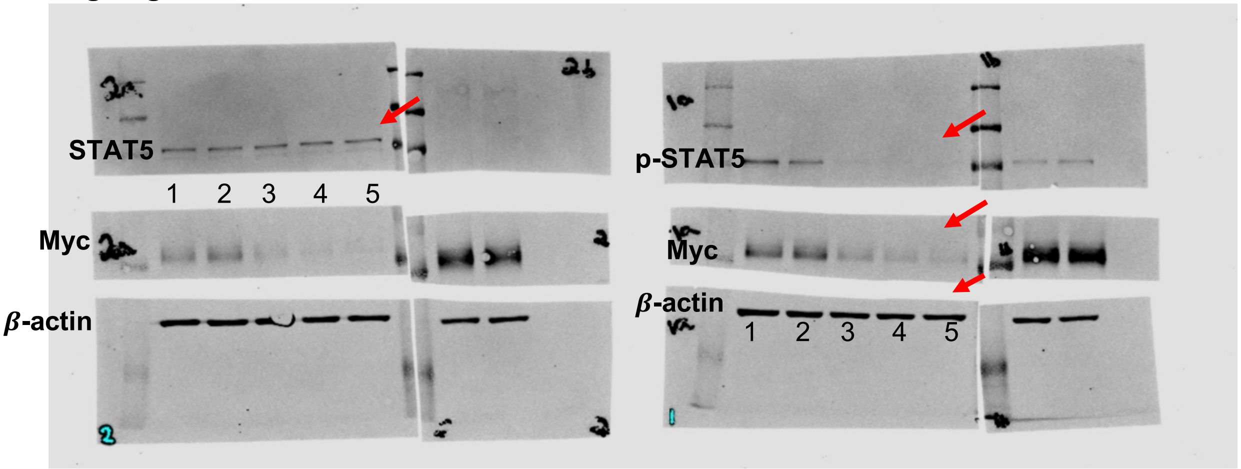

1. MOLM-13 cells with DMSO
2. MOLM-13 cells with 1 nM Gilteritinib
3. MOLM-13 cells with 5 nM Gilteritinib
4. MOLM-13 cells with 10 nM Gilteritinib
5. MOLM-13 cells with 25 nM Gilteritinib

h. Figure 5c.

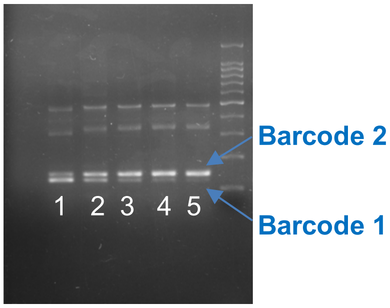

1. **Pre-treatment MV4-11 cells**
2. **MV4-11 cells regenerated from Ara-C treatment at the 'checkpoint'**
3. **MV4-11 cells regenerated from Ara-C treatment 10 days after 'checkpoint' – labelled 'endpoint'**
4. MV4-11 cells regenerated from Ara-C treatment 20 days after 'checkpoint'
5. MV4-11 cells regenerated from Ara-C treatment 30 days after 'checkpoint'

i. Figure S2a.

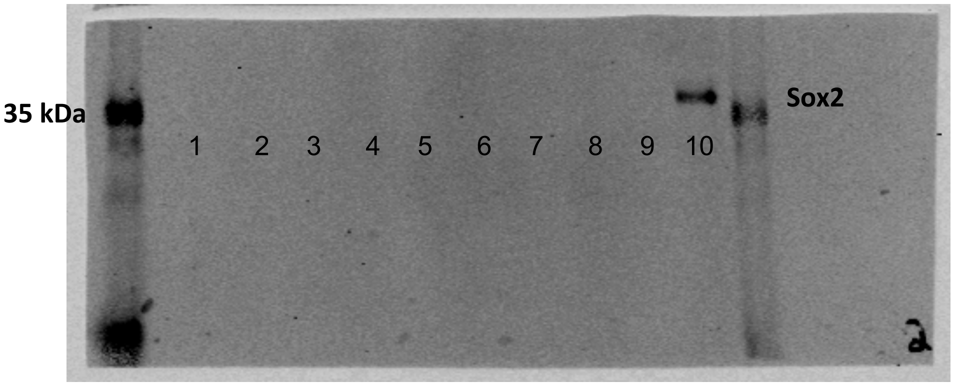

1-9. Initially diagnosed AML bone marrow aspirates
10. SupM2

j. Figure S2b.

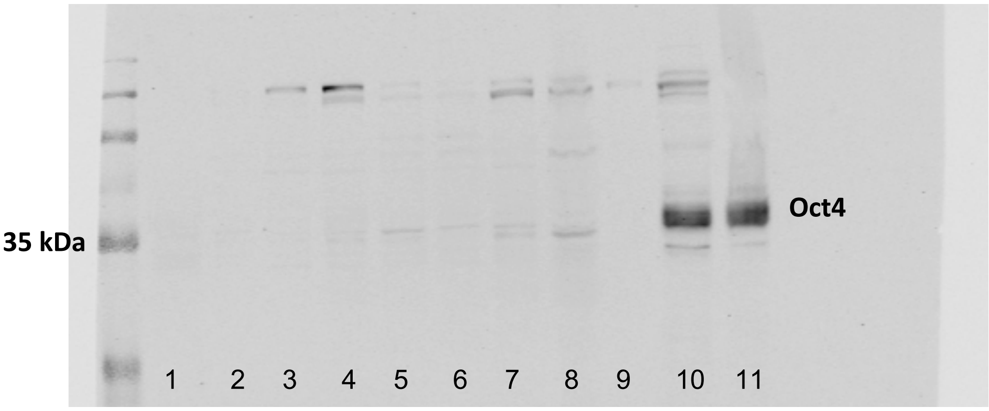

1-9. Initially diagnosed AML bone marrow aspirates
10. Jeko-1 (Mantle cell lymphoma cell line)
11. Mino (Mantle cell lymphoma cell line)

k. Figure S4b.

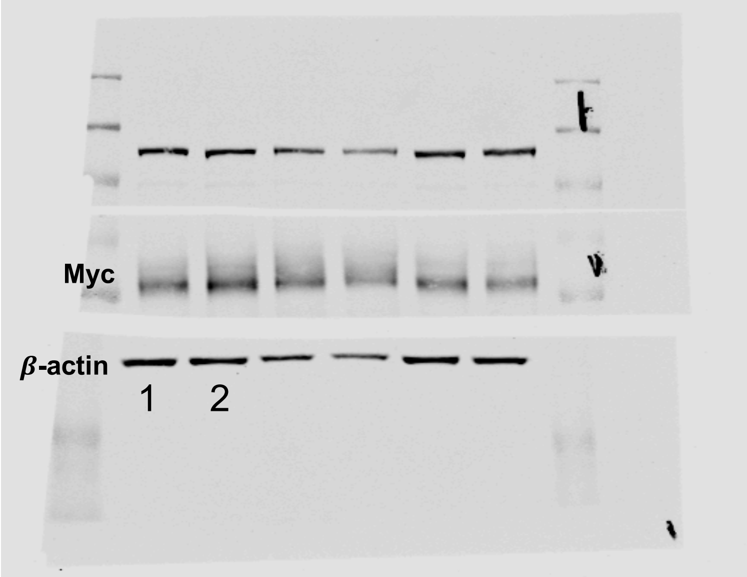

1. MV4-11 SORE6- EV
2. MV4-11 SORE6- Myc overexpression

l. Figure S5c.

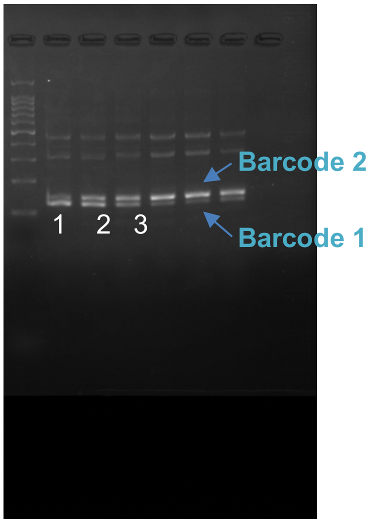

1. Pre-treatment MOLM-13 cells
2. MOLM-13 cells regenerated from Ara-C treatment at the 'checkpoint'
3. MOLM-13 cells regenerated from Ara-C treatment 10 days after 'checkpoint'

Figure S7. Original western blots for (a) Figure 1c, (b) Figure 3a, (c) Figure 3b, (d) Figure 3d, (e) Figure 3f, (f) Figure 3g, (g) Figure 4b, (h) Figure 5c, (i) Figure S2a, (j) Figure S2b, (k) Figure S4b, (l) Figure S5c. If only a portion of the gel was included in the manuscript, then the bolded lanes were included in the paper.
